# Toward standardization, harmonization, and integration of social determinants of health data: A Texas Clinical and Translational Science Award institutions collaboration

**DOI:** 10.1017/cts.2024.2

**Published:** 2024-01-09

**Authors:** Catherine K. Craven, Linda Highfield, Mujeeb Basit, Elmer V. Bernstam, Byeong Yeob Choi, Robert L. Ferrer, Jonathan A. Gelfond, Sandi L. Pruitt, Vaishnavi Kannan, Paula K. Shireman, Heidi Spratt, Kayla J. Torres Morales, Chen-Pin Wang, Zhan Wang, Meredith N. Zozus, Edward C. Sankary, Susanne Schmidt

**Affiliations:** 1 Department of Population Health Sciences, University of Texas Health Science Center San Antonio, Joe R. and Teresa Lozano Long School of Medicine, San Antonio, TX, USA; 2 Division of Clinical Research Informatics, University of Texas Health Science Center San Antonio, Joe R. and Teresa Lozano Long School of Medicine, San Antonio, TX, USA; 3 University of Texas Health Science Center at Houston, School of Public Health, San Antonio, TX, USA; 4 Biostatistics Division, University of Texas Health Science Center San Antonio, Joe R. and Teresa Lozano Long School of Medicine, San Antonio, TX, USA; 5 Department of Internal Medicine, Division of Cardiology, University of Texas Southwestern Medical Center, Dallas, TX, USA; 6 D. Bradley McWilliams School of Biomedical Informatics and Division of General Internal Medicine, University of Texas Health Science Center at Houston, McGovern Medical School, Houston, TX, USA; 7 Department of Community and Family Medicine, University of Texas Health Science Center San Antonio, Joe R. and Teresa Lozano Long School of Medicine, San Antonio, TX, USA; 8 University of Texas Southwestern Medical Center, Harold C. Simmons Comprehensive Cancer Center, Dallas, TX, USA; 9 Houston Methodist, Houston, TX, USA; 10 Department of Surgery, Division of Vascular and Endovascular Surgery, Texas A&M University School of Medicine, Bryan, TX, USA; 11 Departments of Primary Care & Rural Medicine and Medical Physiology, University of Texas Health Science Center San Antonio, San Antonio, TX, USA; 12 Department of Biostatistics and Data Science, University of Texas Medical Branch Galveston, Galveston, TX, USA; 13 University of Texas Health Science Center San Antonio, UT Health Physicians, San Antonio, TX, USA

**Keywords:** Social determinants of health, electronic health records, health information interoperability, health level seven, translational science

## Abstract

**Introduction:**

The focus on social determinants of health (SDOH) and their impact on health outcomes is evident in U.S. federal actions by Centers for Medicare & Medicaid Services and Office of National Coordinator for Health Information Technology. The disproportionate impact of COVID-19 on minorities and communities of color heightened awareness of health inequities and the need for more robust SDOH data collection. Four Clinical and Translational Science Award (CTSA) hubs comprising the Texas Regional CTSA Consortium (TRCC) undertook an inventory to understand what contextual-level SDOH datasets are offered centrally and which individual-level SDOH are collected in structured fields in each electronic health record (EHR) system potentially for all patients.

**Methods:**

Hub teams identified American Community Survey (ACS) datasets available via their enterprise data warehouses for research. Each hub’s EHR analyst team identified structured fields available in their EHR for SDOH using a collection instrument based on a 2021 PCORnet survey and conducted an SDOH field completion rate analysis.

**Results:**

One hub offered ACS datasets centrally. All hubs collected eleven SDOH elements in structured EHR fields. Two collected Homeless and Veteran statuses. Completeness at four hubs was 80%–98%: Ethnicity, Race; < 10%: Education, Financial Strain, Food Insecurity, Housing Security/Stability, Interpersonal Violence, Social Isolation, Stress, Transportation.

**Conclusion:**

Completeness levels for SDOH data in EHR at TRCC hubs varied and were low for most measures. Multiple system-level discussions may be necessary to increase standardized SDOH EHR-based data collection and harmonization to drive effective value-based care, health disparities research, translational interventions, and evidence-based policy.

Since 2020, heightened attention to social determinants of health (SDOH) has been fueled by the national focus on diversity, equity, inclusion, and accessibility [1,2] and the disproportionate impact of the COVID-19 pandemic on minorities and communities of color across the US [3–7]. The National COVID Cohort Consortium (N3C) data led to many research insights demonstrating the power of sharing large-scale electronic health record systems (EHRs) data for secondary research purposes, and the necessity of rich, structured EHR data, including SDOH [8–12]. Yet SDOH is often inconsistently collected in the EHR.

The University of Texas Health Science Center San Antonio (UTHSA) team conducted a regional SDOH inventory given the many streams of effort within health care and research, with federal synergies and convergence pushing toward standardized collection of SDOH data at the point of care within EHRs. UTHSA partnered with the three other Texas institutions also funded by the NIH Clinical and Translational Science Award (CTSA) comprising the Texas Regional CTSA Consortium (TRCC): UT Medial Branch (UTMB) in Galveston, UTHealth Houston (UTH), and UT Southwestern (UTSW) in Dallas. As CTSA hubs, our focus is to develop innovative solutions that will improve the efficiency, quality, and impact of the translation of research and science into practice. As a starting point, the TRCC convened to examine the following questions: (1) What do we need to know about SDOH, regulations, data standards, and uses? (2) Which, if any, contextual SDOH datasets are we making available centrally to researchers via enterprise clinical data warehouses for research? And (3) which individual-level SDOH data are collected in structured fields in each EHR? Our collaborative learning and initial inventory will characterize the environment, and the focus at each institution on SDOH, and inform an ongoing collaboration for harmonization and standardization of collected data elements in the EHR.

## Definitions of social determinants of health and increasing focus on individual-level measures

Definitions of SDOH vary depending on the defining entity’s purview. Some governing entities influence clinical workflows and downstream reimbursement mechanisms; some are data stakeholders in public health programs and supportive services; others may influence intersectional research for addressing vulnerabilities in health disparity populations. A discussion of historical evolution of SDOH definitions can be found in the supplemental materials. The current widely used World Health Organization definition describes SDOH as “the non-medical factors that influence health outcomes. They are the conditions in which people are born, grow, live, work, and age, and the wider set of forces and systems shaping the conditions of daily life. These forces and systems include economic policies and systems, development agendas, social norms, social policies and political systems [13].” The U.S. Department of Health and Human Services (HHS) currently defines SDOH as “the conditions in the environments where people are born, live, learn, work, play, worship, and age that affect a wide range of health, functioning, and quality-of-life outcomes [14].” Although SDOHs have been defined as community and population-level (i.e., contextual) measures, increasingly, individual-level health-related social needs (HRSNs), which include factors such as food insecurity or financial hardship, also are referred to as SDOH [15]. Both types of SDOH together have been explored in recent studies looking at approaches for inclusion of community-level data (e.g., poverty estimates from American Community Survey) in EHRs and in using these data to model at-risk populations with mixed results [15,16]. For example, a resident of a neighborhood with high median income is less likely to have HRSNs compared to a resident of a neighborhood with low median income. However, the association is not sufficient to exclude residents of wealthy neighborhoods from screening [15]. Individual HRSNs are the downstream individual manifestations of aggregate SDOH and are typically assessed via individual patient screening in healthcare settings. In this study, we will use SDOH to mean either type unless specified [17–19]. A discussion of recent U.S. healthcare quality initiatives involving SDOH and EHRs also can be found in the supplemental materials.

## U.S. policy drivers and evolving interoperability standards for increased SDOH data collection in EHRs

The federal Meaningful Use incentive program for hospitals and physician practices mandated almost universal adoption of EHRs starting in 2010. Meaningful Use evolved into Centers for Medicare & Medicaid Services (CMS)’ Medicare Promoting Interoperability Program focusing on information *interoperability*, effective in 2017. The primary standard that vendors incorporate into EHRs for health data computing interoperability, exchange, and integration is Fast Healthcare Interoperability Resources (FHIR®) [20], adopted as of 2012 by Health Level Seven International (HL7), an American National Standards Institute accredited nonprofit standards development organization [21]. The United States Core Data for Interoperability (USCDI) mandated by the Office of the National Coordinator for Health Information Technology (ONC) requires exchange via FHIR of certain individual-level patient data at the data-element level. USCDI is “a standardized set of health data classes and constituent data elements for nationwide, interoperable health information exchange [22].” “These data elements, the most granular level of data to be exchanged, are required for application programing interface certification,” per the ONC (21^st^ Century) Cures Act Final Rule, effective June 30, 2020 [23]. All systems exchanging electronic health data, including EHRs, must make designated data exchangeable at the element level [24].

In May 2019, the Robert Wood Johnson Foundation-funded HL7 Gravity Project began creating FHIR standards for SDOH data. The initial focus was food insecurity, housing instability and quality, and transportation access [25]. SDOH inclusion in the USCDI increased from v1 finalized in June 2020 to v3 in July 2022, which added many additional SDOH elements within the “Patient Demographics/Information” class (e.g., Gender Identity, Sexual Orientation, Tribal Affiliation, Occupation, Occupation Industry) in July 2022. Additional elements explicitly labeled as SDOH are also included in v3 in the “Problems,” “Assessment and Plan of Treatment,” “Procedures,” and “Goals,” data classes, with the class “Health Insurance Information,” added as well. In April 2023, HHS proposed an expansion of the Cures Act, part of which would make v3 the standard within the Certification Program [26]. The many federal agency partners listed in this proposed rule signal coordinated expectations for increased collection of SDOH data within the EHR that will be interoperable. These key stakeholders for data interoperability are driving more SDOH integration in the EHR as they continue to shape the national electronic infrastructure and health information technology landscape.

## Centers for Medicare & Medicaid services reimbursement incentives for increased SDOH data collection to improve healthcare quality and population health

CMS has continued to drive SDOH innovation nationally via its Innovation Center. The center tests new healthcare delivery and reimbursement models including Accountable Care Organizations (ACO; 2012-present) and the Accountable Health Communities Model (AHC; 2017–2023). ACOs recognize the importance of addressing SDOH. However, they have faced “significant difficulties in integrating social services with medical care, lacking data on both their patients’ social needs and the capabilities of potential community partners [27].” These partnerships generally are in early development and “innovation [is] constrained by ACOs’ difficulties in determining how best to approach return on investment, given shorter funding cycles and longer time horizons to see returns on social determinants investments [27].”

AHCs tested standardized, universal offers to screen beneficiaries for individual SDOH needs using the 10-item AHC screening tool [28], coupled with referral to community resources and patient navigation services. AHCs also engaged in community gap analysis and quality improvement strategies to enhance service delivery [29]. Model evaluation is ongoing, and although the AHC screening tool can be deployed in the EHR, there is not a mature standard to guide EHR collection of SDOH data [30,31]. In addition, individual SDOH screening across the US healthcare system remains low, particularly for assessment of multiple co-occurring social needs [19].

## More than a decade of framework development to standardize and structure SDOH data collection – for research and integration in EHRs

Additional nationally used SDOH data collection frameworks are Phenotypes and eXposures (PhenX) toolkit and the Protocol for Responding to & Assessing Patients’ Assets, Risks & Experiences (PRAPARE). The NIH-funded PhenX initiative offered validated measurement protocols of phenotypes and exposures for research since 2007. In 2018, SDOH measures were added, funded by the National Institute on Minority Health and Health Disparities, to inform effective interventions to reduce health disparities [32]. PhenX efforts, however, are not focused on their incorporation within EHRs despite efforts to link EHR data to data collected via PhenX tools [33,34].

In 2013, parallel to and informed by the Institute of Medicine (IOM, now National Academies of Medicine (NAM))-convened group that recommended SDOH data elements for EHR-based collection, a coalition comprising National Association of Community Health Centers, Inc., Association of Asian Pacific Community Health Organizations, and others serving community health, developed PRAPARE. The PRAPARE tool, developed for use in clinical settings, aligned with Healthy People 2020, ICD-10 medical codes, and Meaningful Use Stage 3 quality reporting measures, across 15 SDOH core domains [35]. In 2015, PRAPARE was piloted within multiple widely available EHR systems, including the largest Community Health Center network on a single EHR (Epic) [36]. PRAPARE (17 items + 4 optional questions), CMS’ AHC screener (26 questions), and the two-question Hunger Vital Signs tool are referenced in the USCDI v3 as the structured tool examples via which SDOH assessment data can be collected in the EHR [37].

A 2019 Medicaid survey reported that PRAPARE was the most used standardized SDOH tool in Medicaid Managed Care Organizations (36%) [38]. The AHC screener (29%) was the second most common tool [38]. In total, 50% of Medicaid Managed Care Organizations adopted an existing tool or created their own [38]. Federally Qualified Health Centers, most of which are Medicaid Managed Care Organizations, led the way in SDOH data collection; in the 2017–2018 National Survey of Healthcare Organizations and Systems, nearly one-third of Federally Qualified Health Centers screened patients for 5 SDOH needs: food insecurity, housing instability, utility needs, transportation needs, and interpersonal violence. Overall, ∼24% of hospitals and 16% of physician practices reported screening for all five. Among hospitals, academic medical centers were more likely than nonacademic medical centers to screen patients for those five social needs [39].” Thus, although PRAPARE is available within major EHR systems that does not mean it is enabled or used consistently in a healthcare system.

Major EHR-vendor efforts to incorporate structured fields for longitudinal collection and presentation of SDOH data include the Epic SDOH Wheel, introduced in 2018 [40]. This Wheel, configurable for institutional preferences regarding which SDOHs are documented and which questions are used, allows for more visible, structured documentation of health risk factors such as financial resource strain, transportation needs, alcohol use, depression, intimate partner violence, social connections, physical activity, tobacco use, stress, and food insecurity. The Wheel could help providers or care team members address SDOH and promote care coordination, particularly when integrated with additional Epic applications for population health management (e.g., Healthy Planet) and care coordination (e.g., Compass Rose) [41]. In August 2021, Oracle Cerner introduced Cerner Determinants of Health, a dashboard and supporting set of tools, integrated into the Cerner EHR, to help clinicians collect data, “pinpoint disparities and suggest goals and resources within the patient’s care plan to help target intervention opportunities [42].”

## Materials and methods

### Institutions and team

UTHSA identified and gathered a convergent science multidisciplinary team of 16 experts in biostatistics, clinical and research informatics, demography, disparities research, and medicine from the TRCC institutions (See Table 1).

**Table 1.** Setting characteristics for the four participating Clinical and Translational Science Award (CTSA) health science centers (CTSA hubs)

	UTHealth Houston (UTH)	UT medical branch (UTMB Galveston)	UT health San Antonio (UTHSA)	UT Southwestern (UTSW Dallas)
***Institutional characteristics***				
Carnegie classification of institutions of higher education	R1: Doctoral Universities – Very high research activity equivalent: Special Focus – Research Institution	R1: Doctoral Universities – Very high research activity equivalent: Special Focus – Research Institution	R1: Doctoral Universities – Very high research activity equivalent: Special Focus – Research Institution	R1: Doctoral Universities – Very high research activity equivalent: Special Focus – Research Institution
% Hispanic students per hispanic association of colleges and universities 2020-2021 [43]	Emerging Hispanic Serving Institution	Hispanic Serving	Hispanic Serving	N/A
		Institution At least 25%	Institution At least 25%	
	At least 15% but < 25%			
***EHR and health system characteristics***				
EHR vendor and system version	Epic Systems, Inc. Version May 2022	Epic Systems, Inc. Version May 2022	Epic Systems, Inc. Version November 2022	Epic Systems, Inc. Version November 2021
Year EHR implemented for ambulatory and inpatient	Prev. Allscripts 1990s-early 2000s (ambulatory). Epic since May 2021	2004 (inpatient); 2005 (ambulatory)	2006 (ambulatory); 2024 (inpatient)	2002 (ambulatory); 2008 (inpatient)
Accountable care organization	Established (2013)	Not established	Established (2019)	Established (2017)
Owns hospital	No	Yes	No	Yes
	Contracts physicians to Harris Health and Memorial Herman Systems; does own UT Health Harris County Psychiatric Center, which includes inpatient facility	4 hospital campuses	Contracts physicians to University Hospital System; UT Health San Antonio Multispecialty and Research Hospital is under construction, to open in 2024	
Number of unique patients on date of query	728,436 (Lower patient count due to recent transition to Epic EHR)	3,774,989	2,666,273	1,683,067
***City and county population diversity characteristics***				
City name (2020 U.S. ranking) [44]	Houston (4^th^ largest)	Galveston (Not ranked)	San Antonio (7^th^ largest)	Dallas (9^th^ largest)
County characteristics 2021 1-year ACS estimates [45]	Harris County	Galveston County	Bexar County	Dallas County
County population	4,699,541	350,177	1,986,325	2,568,451
% Latino/hispanic	44.5%	26.5%	62.2%	41.5%
% non-hispanic white alone	26.8%	53.9%	25.0%	26.7%
% Black or African American alone	18.5%	11.6%	7.3%	22.1%
% uninsured	21.8%	13.9%	16.6%	22.0%
% uninsured (1964)	28.6%	18.9%	23.1%	28.3%
% population>65	11.3%	15.1%	12.6%	11.4%
% in poverty	16.4%	11.5%	14.7%	14.3%
% pop ≥ 5 speaks only English	55.5%	80.2%	62.2%	57.2%
% pop ≥ 16 with disability	12.7%	(Not available for 2021)	17.8%	12.7%
% veteran	4.2%	7.3%	10.1%	4.0%

EHR = electronic health record.

### Ethics statement

No IRB approval was necessary because this was not human subjects research, involved no patients or patient data, and comprised institutional resource-related questions only.

#### Centrally available contextual SDOH datasets

Each group completed a spreadsheet listing common sources of contextual (e.g., community and population) SDOH data (e.g., aggregated data from American Community Survey; Food Environment Atlas among others) to indicate which of these, or any other contextual-level datasets, their institution makes centrally available through data warehouse access – regardless of whether these are currently linked/linkable to local EHR data. The spreadsheet was distributed in November and completed in December 2021.

#### Patient-level SDOH data collected in structured fields in the EHR

In December 2021, we finalized a data collection instrument to inventory individual-level SDOH collected in structured EHR fields to be completed by each TRCC hub’s institutional Epic group. We incorporated 20 SDOH data elements that were reported as included in EHRs in results for the “2021 Survey on the Landscape of Collection and Use of SDOH” for institutions participating in the PCORnet clinical research network [46]. The PCORnet survey probed elements derived from recommendations by the PCORI SDOH workgroup, the National Academy of Medicine, CMS, and the Uniform Data System [46]. Based on additional elements that PCORnet survey participants prioritized for EHR-based collection, framework examination, and growing interest in more granular categorization of certain elements for health equity interventions, we added 10 additional elements to our data collection instrument. Table 2 shows the 30 total measures that we analyzed, and questions asked. In addition to “yes” or “no” for structured collection of each listed element, we sought a basic completeness measure: the % of patient records since go-live of the EHR for which there was at least *one* value for each SDOH per patient. We asked whether a framework (e.g., Epic Wheel, PRAPARE) was used within the EHR to collect each element and which ones.

**Table 2.** Individual-level social determinants of health (SDOH) questions and data elements inventoried

*Questions asked for each SDOH Element*					
Collecting this element (as of end 2021) in your EHR in a standardized structured way for “all” patients? Put an “X” if yes.	For those elements you are collecting in a standardized, structured way, are you collecting them via a framework or controlled terminology (e.g., Epic Wheel, PREPARE, Other)? For each, if yes, state which one.	Where yes to structured collection in EHR in 2021, what is the percent of patient records in the EHR with this data in them (i.e., “completed”).	Is your institution going to start collecting this element in a standardized, structured way in the EHR?	Put an “X” next to the five elements that are your health system’s highest priority	If you selected “Other” as one of your health system’s priorities, please comment here on what that “Other” is

*Individual-level SDOH elements inventoried*	*Additional Individual-level elements inventoried*
Language Food Security Education Transportation Employment Status Housing Security/Stability Financial Resource Strain Physical Activity Social Isolation Interpersonal Violence Stress Homeless Status Veteran Status Utilities Household Size Household Income Seasonal/Migrant Farm Worker Neighborhood/Built Environment Discrimination Other	Sexual Orientation Gender Identity (not Sex) Race (NIH categories: Alaska Native, Asian, Black or African American, Native Hawaiian or Other Pacific Islander, and White) More granular race categories than NIH’s Ethnicity (NIH categories: “Hispanic or Latino” and “Not Hispanic or Latino”) More granular ethnicity categories than NIH’s Religion Depression Alcohol Use Tobacco Use

EHR = Electronic Health Record; NIH = National Institutes of Health; PRAPARE = Protocol for Responding to & Assessing Patients Assets, Risks & Experiences.

We assessed completeness of 19 structured fields at each hub. The UTHSA team with their Epic group developed and shared their EHR data query with the other hubs’ Epic groups, who, with minor adjustments, ran it against their EHR data.

## Results

### Centrally available contextual SDOH datasets

We queried which contextual SDOH datasets were centrally available for researchers at each institution (Table 3). Only UTHSA made contextual datasets centrally available (i.e., data warehouse) for all researchers. UTH housed certain SDOH datasets in a research center, but not centrally. At UTSW, an individual researcher (SP) had datasets and shared them with others on request.

**Table 3.** Contextual-level social determinants of health datasets (SDOH; e.g., American Community Survey data): Centralized access at Texas Regional CTSA Consortium (TRCC) institutions for research purposes

UTHealth Houston	No – not available via research data warehouse. However, a specialized individual-level SDOH dataset that had been geocoded with American Community Survey data for an Accountable Health Communities project is available via the research data warehouse and at Center for Health Care Data.
UT medical branch (Galveston)	No - not available via research data warehouse.
UT health San Antonio	Yes - 8 main national data sources loaded into research data warehouse.
UT Southwestern (Dallas)	No - not available via research data warehouse. Limited datasets are curated and made available through an individual researcher (co-author SP) by word of mouth.

CTSA = Clinical and Translational Science Award.

UTHSA had acquired and uploaded eight contextual SDOH datasets into their clinical data warehouse for research with the goal to provide central access for researchers versus the status quo for investigators to acquire and manage these data individually per project. Datasets included four area-level deprivation indices (Area Deprivation Index, Social Vulnerability Index, Distressed Community Index, Social Deprivation Index) as well as contextual information from the County Health Rankings, Air Quality Index, Food Environment Atlas, and various data from the American Community Survey (e.g., poverty rates, median income data).

#### Patient-level SDOH data collected in structured fields in the EHR

Eleven SDOH elements (Table 4) were collected in structured fields at all four institutions: language, food (in)security, education, transportation, employment status, housing security/stability, financial resource strain, physical activity, social isolation, interpersonal violence, and stress. In addition, two additional measures (standard NIH race and ethnicity) were collected in structured fields. Homeless and veteran status were collected by two institutions. Three institutions collected sexual orientation, while two institutions collected gender identity. The hubs also identified four additional relevant elements including religion, depression, alcohol use, and tobacco use. Some elements were collected and displayed in the Epic Wheel at each institution, with the rest collected via other structured fields. None used PRAPARE or the AHC framework.

**Table 4.** Individual-level social determinants of health (SDOH) data elements collected in a structured electronic health record system (EHR) field at each institution

*SDOH Elements Inventoried*	UTHealth Houston	UT Medical Branch (Galveston)	UT Health San Antonio	UT Southwestern (Dallas)
Language	Yes	Yes	Yes	Yes
Food Security	Yes	Yes	Yes	Yes
Education	Yes	Yes	Yes	Yes
Transportation	Yes	Yes	Yes	Yes
Employment Status	Yes	Yes	Yes	Yes
Housing Security/Stability	Yes	Yes	Yes	Yes
Financial Resource Strain	Yes	Yes	Yes	Yes
Physical Activity	Yes	Yes	Yes	Yes
Social Isolation	Yes	Yes	Yes	Yes
Interpersonal Violence	Yes	Yes	Yes	Yes
Stress	Yes	Yes	Yes	Yes
Homeless Status	Yes	x	x	Yes
Veteran Status	Yes	x	x	Yes
Utilities	x	x	x	x
Household Size	x	x	x	x
Household Income	x	x	x	x
Seasonal/Migrant Farm Worker	x	x	x	x
Neighborhood/Built Environment	x	x	x	x
Discrimination	x	x	x	x
***Additional Elements***				
Sexual Orientation	Yes	x	Yes	Yes
Gender Identity (not Sex)	x	Yes	x	Yes
Race (NIH categories: Alaska Native, Asian, Black or African American, Native Hawaiian or Other Pacific Islander, and White)	Yes	Yes	Yes	Yes
More granular race categories than NIH’s	x	x	x	x
Ethnicity (NIH categories: “Hispanic or Latino” and “Not Hispanic or Latino”)	Yes	Yes	Yes	Yes
More granular ethnicity categories than NIH’s	x	x	x	x
Religion	Yes	x	Yes	Yes
Depression	Yes	x	Yes	Yes
Alcohol Use	Yes	x	Yes	Yes
Tobacco Use	Yes	x	Yes	Yes

NIH = National Institutes of Health.

**Figure 1.**
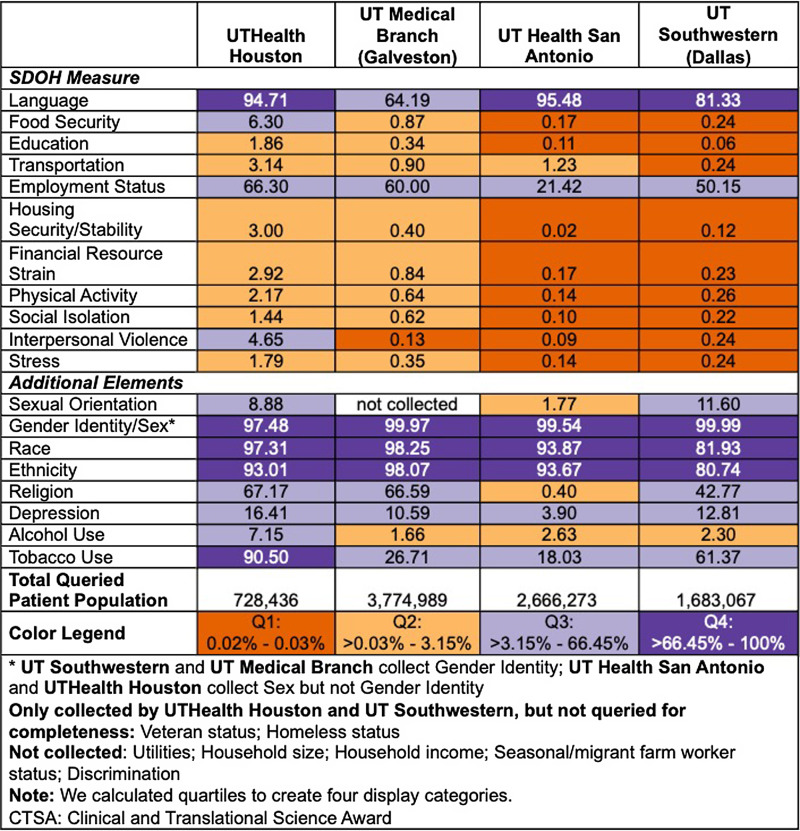
Percent complete of individual-level social determinants of health data elements collected in a structured field in the electronic health record system at four participating Clinical and Translational Science Award institutions.

Availability of structured fields in the EHR, however, is no guarantee of use or completeness of those fields in practice and does not account for SDOH captured in notes. We assessed completeness of 19 structured fields for the four participating CTSA institutions (Fig. 1). Percent complete is based on 728,436 (UTHealth Houston), 3,774,989 (UTMB Branch (Galveston)), 2,666,273 (UT Health San Antonio), and 1,683,067 (UTSW (Dallas)) unique patients. Completeness ranged from 0.02% to over 99.99%. Out of 19 elements queried, all institutions had between 10 and 13 elements with < 10% completeness, including education, financial resource strain, food insecurity, housing security/stability, and transportation – important SDOH measures. The completeness rate for employment status ranged from 21.4% (UTHSA) to 66.3% (UTH), while completeness rate for language ranged from 64.2% (UTMB) to over 95% (UTHSA). Gender identity/sex was over 97% complete at all institutions, while completeness for sexual orientation was higher at UTSW and UTH compared to UTHSA (11.6% and 8.88% vs. 1.77%; UTMB does not collect sexual orientation).

## Discussion

Our inventory characterized the SDOH collection and integration maturity level at each TRCC institution. Next, we discuss the following: how our TRCC inventory results relate to the national landscape; evolving federal context and considerations for maturation of SDOH integration efforts; potential clinical operations and research gains through further SDOH integration; SDOH considerations for researchers; next steps for harmonized SDOH decision making; and conclusions.

### Contextual SDOH dataset integration – early stages at TRCC and nationally

Only UTHSA is making contextual SDOH datasets centrally available for all researchers in its clinical data warehouse for research as of fall 2021. Evolving maturity in this area for the TRCC is in alignment with PCORnet survey responding institutions. Many indicated that integrating and using contextual-level (i.e., community) SDOH data is a near-term priority as part of a more expansive approach to SDOH data collection [47]. Their reasons, like UTHSA’s, included “community health needs assessments and making neighborhood-level data available for research [47].”

The authors discussed data needs and potential research and analytics efficiencies gained by centrally offering such datasets. We also discussed the need to geocode patient addresses from EHR data within the data warehouse to link them to many of these aggregate sets to make the datasets most meaningful/actionable locally/regionally, a process that can be challenging given patient-data issues (e.g., accuracy or missingness of addresses, including for patients without a permanent residence) [48–51]. UTHSA is currently developing a process for geocoding EHR data. We also discussed additional related considerations. These include that institutions need dedicated funding for experts to maintain these datasets centrally; new sets are released from source agencies every few years, and regular geocoding would be necessary to ensure accurate, up-to-date linkage with the latest datasets and patient addresses, which change over time. Resources are also needed to communicate to the research community at each institution the existence of and key details surrounding the access process and use of such centrally available SDOH datasets.

Full integration of contextual-level SDOH datasets into research data warehouses is in the early stages of maturity, but for many research institutions integration will be valuable. Given the resources necessary, institutional leadership should work with their clinical research informatics and research data warehouse experts to formulate integration and sustainability plans.

### Individual-level SDOH data – high interest, low maturity for EHR-based collection, integration, and use

According to PCORnet’s survey, the SDOH elements most commonly collected in structured EHR fields by their respondents included language collected by 64% of PCORnet survey respondents, food security (47%), education (47%), transportation (42%), employment status (42%), housing security (40%) and financial resource strain (38%) [46]. The PCORnet survey did not ask for completeness assessments at responding sites for these items. The TRCC hubs aligned with PCORnet survey institutions. Each TRCC hub collected these elements in its EHR.

Interestingly, two of our four institutions (UTH and UTSW) collected homeless or veteran status, which is twice the 1-in-4 rate reported among PCORnet survey respondents [46]. Because only two of our institutions collected homelessness or veteran status, we did not assess completeness of these measures. Yet, the percentage of veterans in our institutions’ county locations ranged from ∼ 4% (Dallas County and Harris County) to 10.2% (San Antonio – Bexar County). Because of this finding, UTHSA is now working on a structured EHR approach to begin collecting military and veteran status.

In addition, we found great variability in completeness (at least one value per patient) at four CTSA hubs. Further, only three out of the 19 assessed measures had completeness of over 80% (race, ethnicity, and sex/gender identity) at all four hubs. The PCORnet survey highlighted that all participating institutions acknowledged the importance of SDOH data elements, and many health systems had plans to expand and enhance structured EHR collection of SDOH data. The PCORnet survey identified priority data elements to include food security, housing stability, financial resource strain, transportation needs, and education (mentioned by 51%, 49%, 42%, 40%, and 33% of their participants, respectively) [46]. In our study, housing instability, education, transportation, financial resource strain, and food security were collected by all sites. Although these structured fields exist, completeness of these five elements was less than 7%, indicating significant opportunity to improve data collection across all four institutions.

Our findings are similar to other reports as well. A recent national-level analysis showed rates of screening and EHR-based SDOH data collection vary widely, with healthcare institutions that serve higher proportions of underserved patients screening at higher rates than others [39]. Another national survey, published in February 2023 by the American Health Information Management Association (AHIMA), reported that just ∼ 60% of respondents integrated any SDOH data into the EHR [52].

Our findings and other national reports indicate progress and high interest in individual-level SDOH data collection, integration, and use, but with much work remaining. This is true at TRCC hubs where our inventory results will be used in future work to drive SDOH integration and use discussions and decision-making within each hub and across the consortium.

### Evolving context and considerations for CTSAs and other healthcare institutions for maturation of SDOH efforts

Through our examination of the literature and discussions, we identified a number of important evolving factors to inform our continuing SDOH work, which we detail here.

An increasing focus on SDOH integration and use is evident in recent changes to reimbursement models and quality reporting intended to effect widespread healthcare change. Drivers of SDOH screening completeness include exposure to delivery system reform initiatives including innovation models, bundled payments, and commercial ACO contracts [39,53]. Until recently, reimbursement models did not require or pay for SDOH data collection, which provided little incentive to systematically collect such data. SDOH data-driven institutional maturation for care coordination and population health management is required in the latest value-based ACO model “Realizing Equity, Access, and Community Health” status [54,55]. SDOH, in fact, was associated with 38% of variation in Medicare costs between counties [56]. A 2023 EHR-based study at 21 Community Health Centers showed that SDOH influenced care in 35% of surveyed encounters [57]. Given these sizeable impacts, standardized EHR-based collection of SDOH data will benefit stakeholders. Additionally, it can reduce data collection redundancies at the point of care through clinical enterprise policy (e.g., how often SDOH screeners are offered via the patient portal).

Signaling future impact on reimbursement requirements, in April 2022, CMS released the updated 2022–2032 Health Equity Framework [54]. It aligns with other HHS initiatives including the “Healthy People 2030 Framework,” which revised and expanded the SDOH Framework described in its predecessor “Healthy People 2020 [14].” Priority 1 of the CMS Framework called for expansion across healthcare settings of the “collection, reporting, and analysis of standardized data for comprehensive, interoperable, standardized individual-level demographic and SDOH data, including race, ethnicity, language, gender identity, sexual orientation, disability status, and SDOH [54].” It discussed economic stability, educational access and quality, healthcare access and quality, neighborhood and built environment, and social and community context[14,54], and stated that “SDOH data can include information on health literacy, social isolation, housing insecurity, food insecurity, geography, and more [54].”

Quality and accreditation organizations are also emphasizing SDOH integration. In February 2023, National Quality Forum joined National Committee for Quality Assurance and the Joint Commission in “the Sync for Social Needs coalition with the aim of integrating digital SDOH data for standardized exchange across health records using the FHIR standard [58].”

SDOH integration and use maturation will catalyze clinical operations and research improvement gains in many ways. Actionable, standardized, EHR-based SDOH collection will offer rich, robust data for (1) mature analytics for clinical operations; (2) real-time clinical predictive analytics feeding back into EHRs for clinical decision support [3,59–61]; (3) secondary research use internally and for multi-site sharing, including for point-of-care trials and observational studies; (4) machine learning/artificial intelligence research leveraging “big data” [62,63]; and (5) more accurate SDOH profiles for new healthcare and health equity interventions to inform and drive policy changes [14,54,64,65]. All are necessary components of mature, full-cycle translational science in a learning health system [61,66–71].

To realize these gains, institutional strategies need to be updated about what SDOH will be collected and via what structured fields/sections of the EHR [52,62]. Strategies must address workflow considerations such as collection upon patient registration (e.g., via MyChart or in person at kiosks and on tables in clinics), with or without assistance from navigators, and frequency (e.g., annually or more often) [29,30,72]. The AHC or PRAPARE frameworks [30,52] for structured EHR-based SDOH collection are key starting points for minimal, standardized collection, with additional questions added for some measures depending on local populations/needs [30]. At CTSAs, joint effort between the clinical and research leaders is needed to discuss strategies, EHR data capture approaches, and change management efforts for clinical processes, with appropriate patient health information and policy input (e.g., Health Information Management and Compliance). Relying on busy clinicians to collect these data within already over-loaded clinical encounters is not likely to be successful, so adequate staffing to do this is necessary. Such conversations are initiated now at our TRCC institutions resulting from this work.

A number of barriers impact SDOH data collection efforts that should further inform institutional policies. In 2019 and 2021, patients and caregivers who completed SDOH screening in clinical settings found screening acceptable [31,73–75]. However, when asked to consider how their community would view screening, concerns of patient privacy, stigmatization, shame [75], and need for trust with screening personnel arose [73–75] as did perceived risk of bias from providers. Patient answers suggested that screening needs to be conducted with empathy, and cultural and geographic sensitivity, especially with indigenous communities [72]. No small part of the necessary geographic sensitivity relates to the widely varying type and level of resources available in any given location. Patients expressed desire for screening results to be confidential and concerns about the data being shared outside the healthcare team [73]. Data-sharing concerns are particularly pertinent for CTSAs and researchers who require access to SDOH data for research. Strategies to reduce privacy concerns include having trained clinical staff with communication and empathy skills conduct the screening, identifying staff motivation and readiness to conduct screening to reduce bias [31,73,74], and treating the data as a temporary, not permanent, reflection of a patient’s current life status.

Even when SDOH are collected via structured EHR fields in clinical settings, a major barrier to obtaining measurable outcomes from SDOH screening and intervention is that most community-based organizations (CBOs) to which clinical care-services teams refer patients for services and resources to address social needs do not have EHRs. As such, an electronic closed-loop referral process with bidirectional data standards-driven data exchange direct to or accessible via EHRs is rare, and necessitates extensive CBO and community engagement; a group at the University of Texas at Austin has developed a promising technical and engagement approach [27,76–78]. Lack of funding and time needed for multi-stakeholder efforts are challenges we have faced already. Additionally, AHIMA called for federal policy to provision “funding, technical resources, and infrastructure to support coordination and connectivity at the state and local level between healthcare organizations and CBOs [52].” On November 16, 2023, the Biden Administration released the first ever “U.S. Playbook to Address Social Determinants of Health,” which addresses these barriers [79]. In three pillars, the playbook outlines and heralds policy changes to come for (1) advancing SDOH data gathering and interoperability for sharing it; (2) supporting flexible funding to address social needs; and (3) supporting the development of community backbone organizations to electronically link healthcare systems to community-based organizations [80].

### Evolving SDOH context and considerations for researchers

Paucity of SDOH data for many measures from structured EHR-based collection and attrition affecting EHR data means that translational researchers will face analysis challenges, especially in the near term. Given SDOH missingness in the EHR, the analysis of these data will be more complex and difficult to interpret. Further, a systematic review of SDOH EHR data quality issues and how they affect analysis found high risk of bias with 62% of studies examined reporting bias concerns when testing data or evaluating sub-group representation; 21% of studies reported finding SDOH data were missing *not* at random, a concern for all imputation methods [49]. In health disparities research, a clearly articulated statistical estimate is key to correctly mapping the role of each SDOH variable in the analysis model For instance, per NAM, the between-group difference in allowable SDOH covariates should be adjusted away by matching or weighting to ensure unbiased estimands [81,82]. Finally, CTSAs must assess changes that will impact collection and analyses, such as changing categories for sexual identity and gender identity, and for race and ethnicity, under federal review for major expansion for the first time since 1997 [83]. Such changes are necessary given changing demographics and growing understanding of the complexity of relevant societal constructs [84–86].

## Next steps and conclusion

TRCC collaboration’s next steps may include (1) internal discussions about centralization of community-level SDOH datasets; (2) undertaking a robust EHR-field and quality evaluation beyond completeness; (3) examining utility and priority of different measures across TRCC institutions while growing our understanding of how our contextual and individual SDOH data predict health outcomes in our unique populations; (4) developing a cross-institutional governance body to harmonize decisions surrounding a data collection framework, a minimum and sufficient SDOH set for clinical and secondary purposes, and additional customizations; (5) updating policies for uniform SDOH data collection for all patients; and (6) critically, identifying what funding sources will support us and allied stakeholders, including CBOs, in this work. Toward this work, UTHSA’s Chief Health Information Officer is initiating a UT system-level discussion to harmonize decision-making on collection of SDOH data elements in EHR systems. SDOH information is required for effective value-based care, health disparities research, translational interventions, and evidence-based policy in a learning health system. Thus, as translational research leaders, CTSA institutions are ideally positioned to develop, evaluate, and implement common standards and practices to collect comparable SDOH information.

## Supporting information

Craven et al. supplementary material Craven et al. supplementary material

## Supplementary material

The supplementary material for this article can be found at https://doi.org/10.1017/cts.2024.2.
