# Toward standardization, harmonization, and integration of social determinants of health data: A Texas Clinical and Translational Science Award institutions collaboration – CORRIGENDUM

**DOI:** 10.1017/cts.2024.516

**Published:** 2024-04-18

**Authors:** Catherine K. Craven, Linda Highfield, Mujeeb Basit, Elmer V. Bernstam, Byeong Yeob Choi, Robert L. Ferrer, Jonathan A. Gelfond, Sandi L. Pruitt, Vaishnavi Kannan, Paula K. Shireman, Heidi Spratt, Kayla J. Torres Morales, Chen-Pin Wang, Zhan Wang, Meredith N. Zozus, Edward C. Sankary, Susanne Schmidt

The above article was published with two small typos in the color legend for Figure 1. Please see below for the corrected figure.

**Figure 1.**
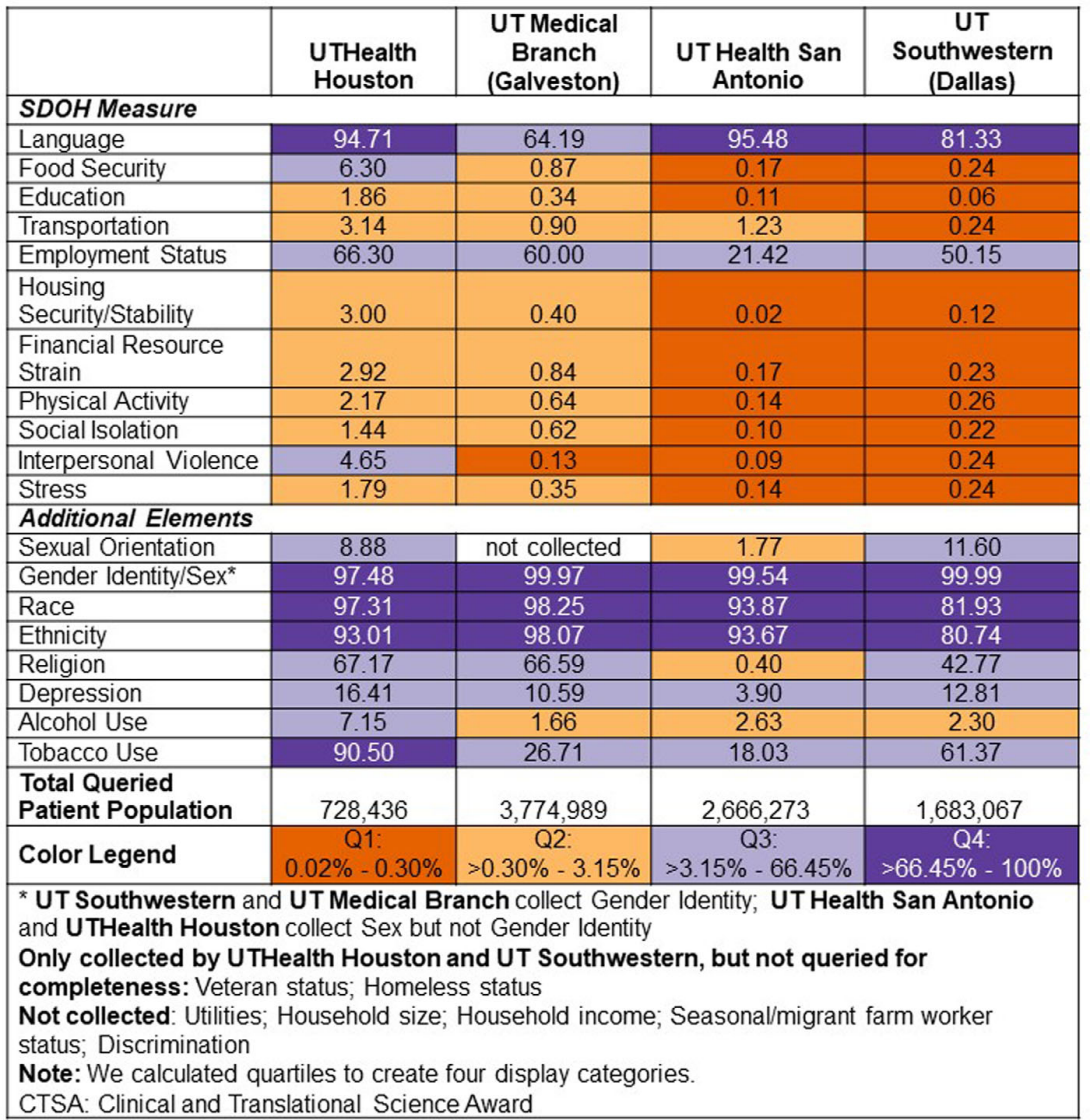
Percent complete of individual-level social determinants of health data elements collected in a structured field in the electronic health record system at four participating Clinical and Translational Science Award institutions.
