# Supplementary material for: Toward standardization, harmonization, and integration of social determinants of health data: A Texas Clinical and Translational Science Award institutions collaboration
Source: J Clin Transl Sci. 2024 Jan 9;8(1):e17. doi: 10.1017/cts.2024.2 (PMC10880009; doi:10.1017/cts.2024.2)
Supplement: Craven et al. supplementary material [file S2059866124000025sup001.docx]

**Supplemental Materials**

**Historical Considerations of Social Determinants of Health (SDOH)**

Scientific exploration of social factors in health disparities is rooted in pioneering empiric public health inquiry particularly related to infectious diseases.^1-5^ In the second half of the 20^th^ Century, epidemiology shifted focus from social causation to individual-level risk factors, and in 1999, chronic diseases outstripped infectious diseases as the world-wide most common causes of death.^4,6^ However, it is not an either/or situation; it is both. Almost 80% of chronic disease is linked to four individual behaviors: tobacco use, inadequate physical activity, unhealthy diet, and alcohol misuse patterns.^4,7^ Yet these behaviors “display social patterning” with the first three reported more frequently among socially disadvantaged groups,^8^ and the fourth, in the form of binge drinking, more common among higher status individuals.^4,9^ World Health Organization (WHO) states: “Research shows that the social determinants can be more important than health care or lifestyle choices in influencing health. Numerous studies suggest that SDOH account for between 30-55% of health outcomes, and estimates show that the contribution of sectors outside health to population health outcomes exceeds the contribution from the health sector.”^10^

**Recent U.S. healthcare quality initiatives involving SDOH and EHRs**

Significant recent U.S. healthcare initiatives have been designed to catalyze collection of behavioral and social factors data to inform individual patient care and population health management, particularly for chronic conditions. The HITECH Act within the 2009 American Recovery and Reinvestment Act created the Meaningful Use (MU) incentive program for hospitals and physician practices, which almost universally implemented EHRs, with reimbursement for EHR investments dependent on phases of reporting of care quality measures generated from patient data collected within the EHR.^11^ The major MU foci were use of certified EHR in a meaningful manner (e.g., e-prescribing), electronic exchange of health information to improve health care quality, and submission of clinical quality measures to Centers for Medicare & Medicaid Services (CMS).^11^ Notably, at both hospitals and physician practices, smoking status was one of the required core MU measures. In 2014, because of the centrality of SDOH in health outcomes, the National Academies of Medicine (NAM; formerly the Institute of Medicine (IOM)) published the report “Recommended Social and Behavior Domains and Measures for Electronic Health Records: Phase 2.” NAM recommended 12 measures from 11 patient-reported domains, four of which are routinely collected in clinical settings (tobacco and alcohol use, race/ethnicity, and residential address) and 1 neighborhood/community-level domain, each to be answered by 1-4 questions.^12^ The report, requested by U.S. National Institutes of Health (NIH) Office of Behavioral and Social Sciences Research (OBSSR), with the U.S. Centers for Disease Control and Prevention (CDC), CMS, the Substance Abuse and Mental Health Services Administration, the Department of Veteran Affairs, the Robert Wood Johnson Foundation (RWJF) and other large organizations,^12^ stated that these social and behavioral measures should be collected in EHRs. The intent was to influence certification of EHRs by the U.S. Office of the National Coordinator for Health Information Technology (ONC) and MU (Stage 3) objectives for quality reporting to CMS. The report emphasized the necessity of standard measures for capturing these data and for *exchange* of these data via data interoperability standards. The report also emphasized that the addition of social and behavioral data to EHRs will “enable novel research, and the impact of the research is likely to be greater if guided by federal prioritization activities.“^12^ Accordingly, the report recommended that the NIH Office of the Director “develop a plan for advancing research using social and behavioral determinants of health in electronic health records’ and that OBSSR coordinate this plan with the input across the NIH institutes and centers.”^12^ Additionally, the report recommended continued Health and Human Services (HHS) review of advances in the measurement of social and behavioral determinants of health and recommendations for new standards and data elements for inclusion in EHRs.

References

1. Mackenbach JP. Politics is nothing but medicine at a larger scale: reflections on public health’s biggest idea. *Journal of Epidemiology and Community Health*. 2009;63(3):181-184. doi:10.1136/jech.2008.077032

2. Julia C, Valleron AJ. Louis-Rene Villerme (1782-1863), a pioneer in social epidemiology: re-analysis of his data on comparative mortality in Paris in the early 19th century. *J Epidemiol Community Health*. Aug 2011;65(8):666-70. doi:10.1136/jech.2009.087957

3. Engels F. The condition of the working class in England. 1845. *American journal of public health*. Aug 2003;93(8):1246-9. doi:10.2105/ajph.93.8.1246

4. Ferrer RL. Social Determinants of Health. In: Daaleman TP, Helton MR, eds. *Chronic Illness Care: Principles and Practice*. Springer International Publishing; 2018:435-449.

5. Acheson ED. Edwin Chadwick and the world we live in. *Lancet*. Dec 15 1990;336(8729):1482-5. doi:10.1016/0140-6736(90)93186-s

6. World Health Organization. The World health report : 1999 : Making a difference. Geneva: World Health Organization; 1999.

7. Malter Cohen M, Jing D, Yang RR, Tottenham N, Lee FS, Casey BJ. Early-life stress has persistent effects on amygdala function and development in mice and humans. *Proceedings of the National Academy of Sciences*. 2013;110(45):18274-18278. doi:doi:10.1073/pnas.1310163110

8. Gerth HH, Mills CW. *From Max Weber. Essays in sociology*. Routledge; 2009.

9. Turner RJ, Wheaton B. Checklist measurement of stressful life events. In: Cohen S, Kessler RC, Gordon LU, eds. *Measuring stress: A guide for health and social scientists*. Oxford University Press; 1995:29-58.

10. World Health Organization. Social Determinants of Health. Accessed 05/27/2023, 2023. <https://www.who.int/health-topics/social-determinants-of-health#tab=tab_1>

11. Centers for Medicare & Medicaid Services (CMS). Medicare & Medicais EHR Incentive Program - Meaningful Use Stage 1 Requirements Overview. Accessed 05/28/2023, 2023. <https://www.cms.gov/Regulations-and-Guidance/Legislation/EHRIncentivePrograms/downloads/mu_stage1_reqoverview.pdf>

12. Committee on the Recommended Social and Behavioral Domains and Measures for Electronic Health Records, Board on Population Health and Public Health Practice, Institute of Medicine. *Capturing Social and Behavioral Domains and Measures in Electronic Health Records - Phase 2*. National Academies Press (US); 2015. <https://www.ncbi.nlm.nih.gov/books/NBK268995/>
